# Rare presentation of an isolated bilateral cerebral peduncular infarction: A case report and review of the literature

**DOI:** 10.1097/MD.0000000000017665

**Published:** 2019-11-01

**Authors:** Xiaoxue Fu, Hongyu Li, Xiaoquan Tian, Wei Wang, Hong Liu

**Affiliations:** a Department of Neurology, Heping Hospital Affiliated to Changzhi Medical College, Shanxi Province; b Beijing Zhendong Guangming Pharmaceutical Research Institute Co Ltd, Beijing, China.

**Keywords:** ataxia, bilateral cerebral peduncular infarction, dysarthria, mild paresis of the extremities, sensory disturbance

## Abstract

**Rationale:**

Due to the rarity of bilateral cerebral peduncular infarction (BCPI), its symptoms and prognosis are not clear. It is necessary to collect cases of pure cerebral peduncular infarction, explore the etiology and anatomy of midbrain infarction in depth, and develop meaningful tools for explaining clinical symptoms and predicting prognosis of patients.

**Patient concerns:**

We here provide a case of isolated BCPI with uncommon symptoms of ataxia, dysarthria, sensory disturbance, normal muscular strength, and full eye movements.

**Diagnoses:**

Diffusion weighted images and apparent diffusion coefficient map of our patient revealed acute and isolated bilateral peduncle cerebrum infarction.

**Interventions:**

Drugs that could improve circulation and antiplatelet were used in therapy.

**Outcomes:**

The infarct size was enlarged and new infarction was identified in the splenium of the corpus callosum and pons. The patient developed progressed disorder of consciousness and died at the eleventh day.

**Lessons:**

We inferred that the symptoms of ataxia, dysarthria, sensory disturbance, and mild paresis of the extremities could be prominent features of patients with pure cerebral peduncular infarction. We hypothesize that pure BCPI is also related to severe basilar artery stenosis or occlusion and there is no collateral circulation from PCA. This may explain the corresponding distribution of cerebral peduncular infarction and its poor prognosis. For these reasons, exploring etiology and anatomy of midbrain infarction in depth would have clinical value for predicting symptoms and prognosis.

## 1 Introduction

The prevalence of bilateral cerebral peduncular infarction (BCPI) is unknown because of the limited available literature. However, it has been reported that the incidence of isolated midbrain infarction varies only from 0.7% to 2.3%.^[1]^ Data from one medical center showed that BCPI accounts for 0.26% of all admitted patients with ischemic stroke.^[2]^ Chen et al assessed 14 cases with BCPI, but they were not pure midbrain infractions, and other sites of infarction were found in the thalamus, pons, and cerebellum.^[1,3]^ Asakawa et al^[4]^ also reported a BCPI that simulated a Mickey Mouse ears sign but was also accompanied by infarction of the cerebellum. Here, we identified such a case of isolated BCPI with uncommon symptoms of ataxia, dysarthria, sensory disturbance, and normal muscular strength and full eye movements. Before our report, only Zhou et al^[5]^ had described any case of isolated BCPI without locked-in syndrome. Interestingly, two cases had almost the same symptoms, with a few differences. For this reason, we here discuss the cause of these symptoms in this case and its possible etiology by comparing it to other related cases.

## 2 Case report

**Figure 1:**
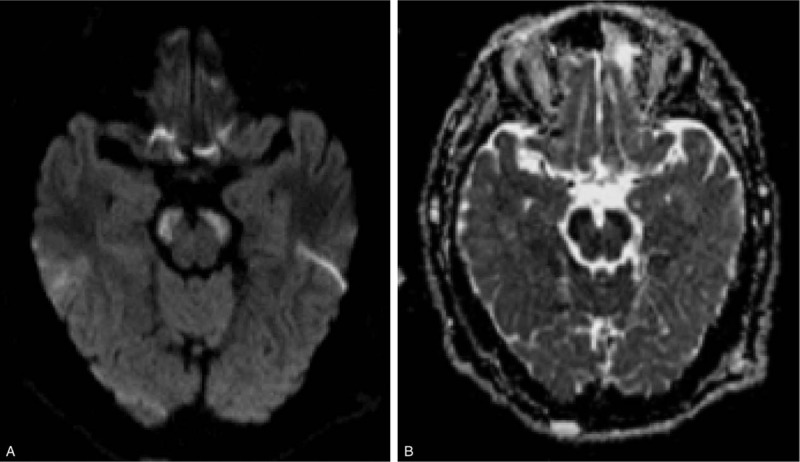
(A). Diffusion weighted imaging (DWI) performed upon admission showed a hyperintense bilateral peduncle cerebrum infarction; (B). With slight hypointensity in the apparent diffusion coefficient (ADC) map at the same position.

**Figure 2:**
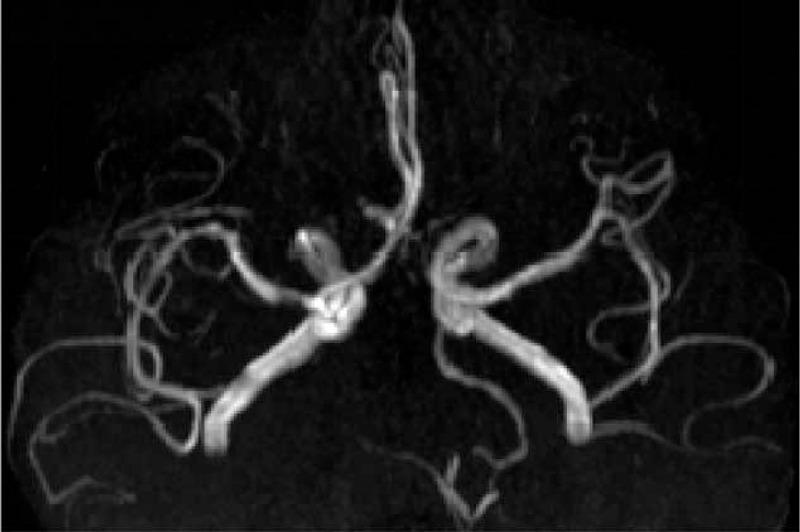
Magnetic resonance angiography (MRA) showed the vertebrobasilar artery and right posterior cerebral artery (PCA) occlusion, left arteriae cerebral artery (ACA) stenosis, and left fetal posterior cerebral artery (fPCA).

**Figure 3:**
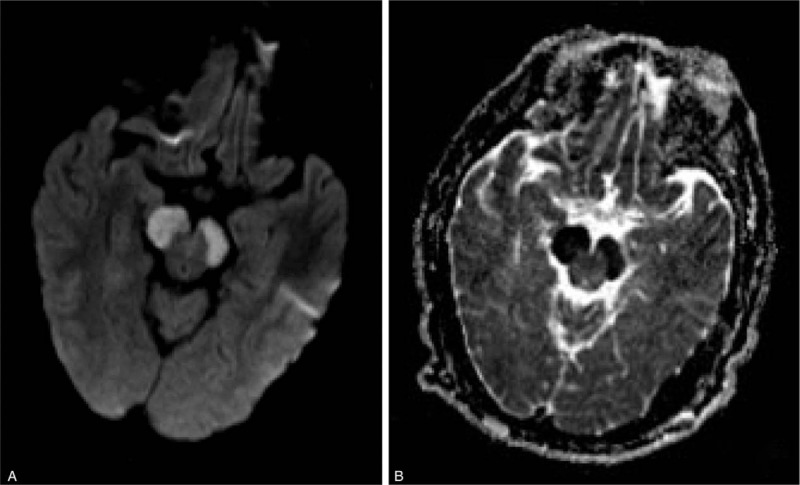
Re-examination of DWI (A) and ADC (B) on the eighth day of hospitalization.

A 51-year-old man came to the Neurological Intensive Care Unit with a 1-day history of dizziness, dysarthria, pseudobulbar paralysis, unsteady gait, and hiccups but without double vision or gaze palsy. His past medical history included a 15-year-hypertension and a 10-year-diabetes mellitus. His family history did not include hypertension, diabetes mellitus, or stroke. He was a smoker with a 2-pack-a-day habit going back 30 years, drank alcohol in moderation, and said he did not use illicit drugs. Upon admission, except for a blood pressure of 201/115 mmHg, his vital signs were unremarkable. Neurological exam showed he had severe dysphagia, ataxia in all of his limbs, but more pronounced on the left side. Sensory disturbance also happened on the left side. The patient showed pathologic reflexes from bilateral Babinski tests. There was no restricted ocular movement, nystagmus, ptosis, or visual deficit. The patient did not exhibit any weakness of limbs. Because of pseudobulbar paralysis, he showed pathological laughing and crying on our examination. Brain computed tomography revealed no evidence of infarction or hemorrhage. Diffusion weighted imaging (DWI) performed upon admission showed a hyperintense bilateral peduncle cerebrum infarction and slight hypointensity in the apparent diffusion coefficient (ADC) map (Fig. 1). Magnetic resonance angiography (MRA) showed the vertebrobasilar artery and right posterior cerebral artery (PCA) occlusion, left arteriae cerebral artery (ACA) stenosis, and left fetal posterior cerebral artery (fPCA) (Fig. 2). Drugs that could improve circulation and antiplatelet were administered for therapy. However, his conscious state deteriorated on the fifth day of hospitalization. On the eighth day, he showed lethargy, severe hypovolemia caused by dyspnea, asthenocoria, horizontal gaze to the left side, horizontal nystagmus, and quadriplegia. We re-examined the MRI and the infarct size was enlarged and new infarction was identified in the splenium of the corpus callosum and pons (Fig. 3). The patient's disordered consciousness worsened, and he died on the 11th day.

## 3 Discussion

We systematically searched case reports of pure cerebral peduncular infarction using PubMed from February 1, 2019. The sole search terms were cerebral peduncular infarction. Upon review of the retrieved articles, cases of pure cerebral peduncular infarction with detailed description of clinical symptoms were included. Finally, only 6 articles (with 12 cases) that fit our inclusion and exclusion criteria were included. We summarized associated symptoms in these articles on cases of pure cerebral peduncular infarction and compared with our case (Table 1).

**Table 1:**
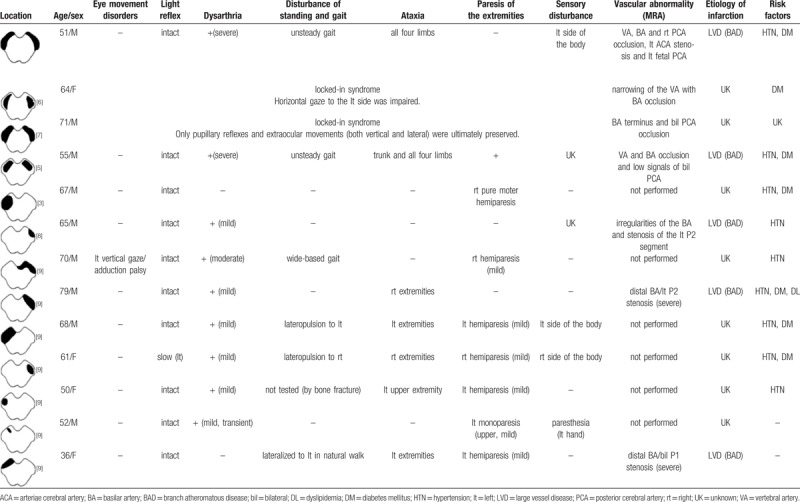
MRI-confirmed reported cases of infarction limited to the cerebral peduncular.

Previously, cases of BCPI involved most of the lateral portion of the peduncle, which were found to have classic Mickey Mouse ears^[4]^ or the traditional Chinese eight character sign^[5]^ on DWI. The same sign was present in our case.

Disordered consciousness and locked-in syndrome (LIS) are common symptoms of BCPI.^[2]^ LIS has been attributed to ventral pontine lesions. However, pure BCPI has also been associated with LIS and vegetative state, but this is extremely rare,^[6]^ with only two cases reported.^[6,7]^

**Figure 4:**
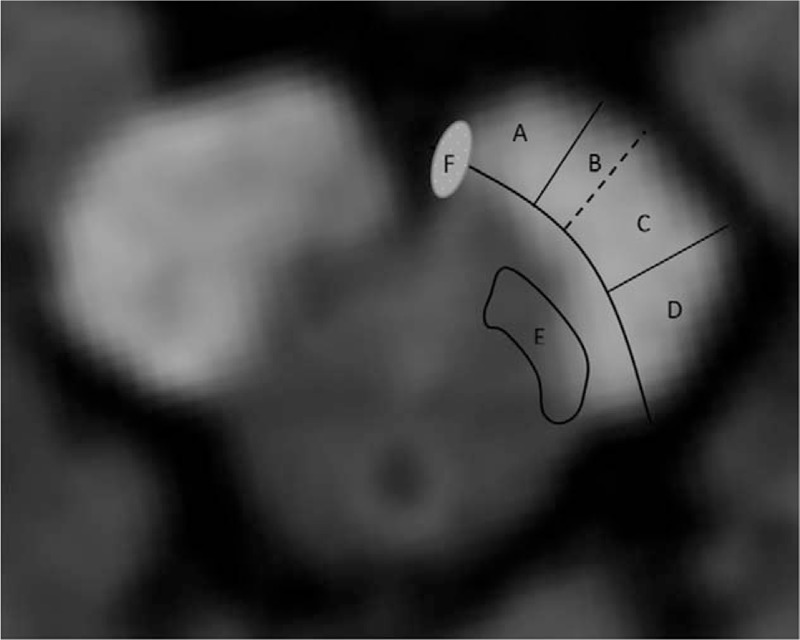
Functional areas of the cerebral peduncule.

We found that the most common symptoms included ataxia, dysarthria, sensory disturbance, and mild paresis of the extremities of pure cerebral peduncular infarction, with few disorders of eye-movement or light reflex appearing upon neurologic examination. The infarction of paramedian area (Fig. 4F area), which includes the oculomotor nerve, is why eye movement disorder occurred in only the one patient. Nerve fibers coming from the parietal lobe and frontal lobe descend to the corticospinal tract (CST) and finally ran in the pedunculus cerebri, reaching the cerebellum via the corticoponto cerebellar tract (CPCT). The damage to the CST (Fig. 4C area) and CPCT (Fig. 4A area) in the pedunculus cerebri are the cause of the mild paresis of the extremities, dysarthria, and ataxia. However, it remains unclear why only mild hemiparesis was present when the pyramidal tract at the crus cerebri was heavily involved. Through analysis of the topography of the infarct, Bogousslavsky et al found that patients with hemiparesis also consistently showed representation of the lower limb in the lateral part of cerebral peduncle, the face and upper limb being represented more medially.^[3]^ The spinothalamic tract located at the dorsolateral part of the medial lemniscus in the lateral area of the midbrain (Fig. 4E). The disturbance of superficial sensory is likely due to the damage to the spinothalamic tract. Similarly, cerebral peduncular infarction also affects the function of the corticonuclear tract (Fig. 4B), tractus parietopontinus, temporopontile tract, and tractus occipitopontine (Fig. 4D), but we did not observe any obvious symptoms. We inferred that the ataxia, dysarthria, sensory disturbance, and mild paresis of the extremities could be features of patients with pure cerebral peduncular infarction. Our patient's symptoms, asymmetric ataxia, hemianesthesia, and normal muscle strength, may suggest that his BCPI was incomplete, and he retained some of his neural functions.

The vascularization of the midbrain is complex because there is a significant contribution by the perforating branches of the posterior communicating arteries and the peduncular perforating arteries and circumflex branches of the precommunical (P1) or P2 segment of the PCA in addition to the supply through basilar and cerebellar arteries. Chen et al^[2]^ found that a decrease in the flow signals of the proximal portions of the P1 segments on the MRA played a crucial role in the occurrence of BCPI. They proposed that perforating branches of the P1 segments plays an important role in cerebral peduncular infarction. By summarizing the MRA results from Table 1, we found that all of the cases more or less shared a problem of the basilar artery (occlusion or stenosis) and most of them also had problems with PCA. We conjecture that pure BCPI may also be related to severe basilar artery occlusion or stenosis and no collateral circulation from PCA. This may explain the corresponding distribution of cerebral peduncular infarction and its poor prognosis.

The patient underwent echocardiography with no evidence of any existing emboligenic heart disease. However, his MRA showed the stenosis and occlusion of the arteries. Ultrasonography confirmed a different degree of carotid atherosclerosis and plaque formation. We have reason to believe the infarct was caused by atherothrombotic large vessel disease (LVD), also called large-artery atherosclerosis (LAA). Uniformly, in another study, LAA was considered the primary cause responsible for 78.6% of BCPI. However, it is unclear whether the infarct associated with BA occlusion is due to large-to-small artery microembolism or to perfusion failure from occlusion of the mouth of branches that originate at the level of the stenosis.

The symptoms of pure cerebral peduncular infarction might be a reproducible finding, although it is rare. It is still necessary to collect more cases of pure cerebral peduncular infarction to explore the etiology and anatomy of midbrain infarction in depth. This would provide information of clinical significance for predicting symptoms and prognosis of patients.

## Author contributions

**Writing – original draft:** Xiaoxue Fu.

**Writing – review & editing:** Hong Liu, Hongyu Li, Xiaoquan Tian, Wei Wang.
